# Isokinetic Dynamometer Leg Extensor Peak Torque Measurement: A Time-Delayed Reliability and Score Selection Analysis Study

**DOI:** 10.3390/jfmk8020062

**Published:** 2023-05-12

**Authors:** Brennan J. Thompson, Jennifer Xu

**Affiliations:** 1 Department of Kinesiology and Health Science, Utah State University, Logan, UT 84322, USA; 2 Movement Research Clinic, Sorenson Legacy Foundation Center for Clinical Excellence, Utah State University, Logan, UT 84322, USA; 3 Department of Kinesiology, University of Virginia, Charlottesville, VA 22904, USA

**Keywords:** Biodex, muscle strength, muscle function, strength testing, peak force, quadriceps

## Abstract

The reliability of isokinetic peak torque (PT) has been reported mostly using a short-term (<~10 day) inter-trial testing time frame. However, many studies and programs utilize a long-term (several weeks to months) inter-trial testing period. Additionally, the methods by which the PT value is selected and reported from a multiple rep testing scheme have not been well investigated for both reliability and PT absolute performance comparisons. The purpose of this study was to investigate the long-term reliability of isokinetic and isometric PT of the leg extensors with an emphasis on the differences among several PT score selection methods. Thirteen men and women (age = 19.5 years) underwent two testing trials separated by 28.8 (±1.8) days. Testing included maximal voluntary contractions of three sets of three reps for two isokinetic contraction conditions of 60 (Isok60) and 240 (Isok240) deg/s velocities, and three sets of one rep of isometric contractions for the leg extensors. The PT score was derived from seven different methods (see text for descriptions). Reliability as assessed from intraclass correlation coefficients (ICCs) varied widely across contraction conditions and PT score selection parameters. The Isok60 velocity overall had lower reliability (ICCs = 0.48–0.81) than Isok240 (0.77–0.87) across the conditions whereas the isometric PT variables showed moderate reliability (0.71–0.73). Overall the set 1 PT score selection parameters were generally lower (*p* ≤ 0.05) than those that involved sets two and three. Systematic error (*p* ≤ 0.05) was shown for 6 out of the 17 PT selection variables. On a subjective interpretation basis, when taking everything into account the best overall combination of time/trial efficiency, reliability, best/highest PT score parameter, and reduced risk of systematic bias appears to be the PT variable that uses the average of the highest two reps of the first two sets of three reps—i.e., averaging the highest two values of the six total reps from the first two sets.

## 1. Introduction

Isokinetic (i.e., constant velocity contractions) strength and/or muscle function testing is commonly implemented across a range of settings—such as sports performance, clinical/rehabilitation, laboratory, and research settings—due to the advantages it has of providing internal, repeatable controls (e.g., standardized force—velocity components of muscle force output, range of motion, testing scheme, etc.) along with its being considered a relatively safe means of testing strength [1,2,3]. Accordingly, it is often considered to be the gold standard assessment for quantifying strength [4,5,6,7,8,9].

Proponents of isokinetic dynamometer testing also point to the reportedly high reliability of its output measures [6,8,10] as an advantageous feature of this type of testing. Dynamometer outputs can provide a multitude of variables, such as torque, power, and velocity. Although muscle power is reported somewhat frequently in isokinetic studies [11], peak torque (PT) is the most commonly reported variable [3,6,7,12] largely because it reflects the most direct, objective, and precise measurement of one’s maximal strength capacity that can be assessed in an applied and yet controlled physiological context. A host of reliability studies on isokinetic dynamometers have been performed over the last several decades for a variety of different populations and these have, for the most part, shown high reliability for the PT variable [3]. However, one common feature of the majority of these studies is that they were designed to assess reliability using a short-term test-retest study design. Short-term reliability uses a test-retest design that tests and then retests the measure of interest (i.e., PT, power, work, etc.) over the course of a several-hour [8,13]-to-several-day (~1 week) inter-trial period [4,8,13,14].

However, in the field of human performance/exercise science, it is common for studies to employ research designs that include an intervention period that spans many weeks to months [15,16]. The commonality of such designs is because the field of study necessitates the substantial use of longitudinal designs that implement a myriad of treatments and interventions (e.g., exercise, nutrition, stretching programs, etc.). Additionally, these “medium-to-long-term” intervention studies often involve research protocols that utilize a control condition or group to match the duration of the study intervention. Few studies have examined the reliability of the isokinetic PT measure in a long-term inter-trial reliability design. Madsen [13] examined knee extensor PT on both short-term (median of 2 and 7 days between trials) and long-term (median of 32 days between trials) and found that the reliability (as measured by the coefficient of variation [CV] statistic) for the PT 240 deg/s variable was worse for the long-term vs. the short-term reliability period. Kannus [6] also examined the isokinetic PT reliability of the knee extensors with a lengthy eight-week inter-trial period and found rather high CV values (CV = 16–20%) reflecting worse than the usually reported reliability for the PT parameter.

These long-term reliability studies showed that isokinetic PT reliability may be impacted negatively by a longer-term (>~2 weeks) inter-trial period. They were also limited in the fact that they only reported CVs for reliability (and not the important intraclass correlations coefficient [ICC] and standard error of measurement [SEM] statistics), had a wide variability/spread for the number of inter-trial days for their long-term period (Madsen’s inter-trial period ranged widely between 23–48 days for the long-term period), and examined a population of nonathletic women belonging to a large age range of 18–52 years [13]. Thus, the test-retest reliability of the isokinetic dynamometer tests pertaining to a long-term inter-trial reliability protocol is not well established especially in different, more homogenous populations using a more comprehensive set of reliability statistics. The use of this approach would put a greater emphasis on the ecological validity of the actual testing scheme commonly implemented by longitudinal studies and may capture effects that have not been previously estimated or appreciated.

Another common disparity in the research is the method for selecting and calculating the PT variable from the multiple trials usually performed. A common isokinetic testing protocol for assessing PT values on an isokinetic dynamometer uses a single set model where the subjects are typically warmed-up with concentric isokinetic contractions (often following a general warm-up), briefly familiarized and then perform 1 set (per velocity) of usually ~3–5 repetitions [2,6,13,16,17,18,19]. Although some studies have used a multiple set [4,20] testing scheme, it remains unknown as to whether the best testing protocol for determining one’s PT performance may involve more than a single set as a potential means to optimize best performance detection and reliability. Indeed, there is some evidence that a multiple-trial muscle function test protocol may be optimal. Callahan et al. [21] compared lower body leg power values between single- and multiple-set models and reported that the five-set approach showed better reliability and higher scores compared to the single-set approach. Their findings suggest a potential advantage for multiple trial testing protocols, although it should be noted that this was based on muscle power using pneumatic resistance equipment in older persons. Nevertheless, the rationale for a multiple trial protocol seems reasonable from the framework of increased potential to identify the best performance. However, there is a trade-off, such that a high number of trials uses valuable investigator and participant time and increases physical and mental effort resources. It may also have a negative (i.e., fatigue) influence on subsequent performances in an intra-day testing scheme that implements a multi-assessment battery.

To further complicate the analysis, the best method for determining or selecting the PT value that best represents the person’s maximal strength is also varied and has not been well studied. For instance, PT has been reported as the single highest value of all sets/reps [4,16,17,18,19,20], as the mean of all values [13,17], and as some variation of dropping one or more values and averaging the rest of the values [2,22,23]. No consensus to date has been provided that offers investigators the most objectively superior procedures for determining how to design an optimal testing protocol and select the best PT value out of the multi-value output. Taken further, the reliability of the different approaches for determining the PT value has not been examined. Understanding the reliability profile of the different PT selection methods would be useful for determining the best approach for analyzing this common variable. Moreover, the different methods for describing PT may likely yield considerably different PT values on an absolute performance basis. Thus, a better understanding of the ideal protocol/methods to report PT would provide an advantage by helping to optimize testing schemes in a way that blends the best use of time/effort, reliability, and acquiring the best performance score.

Therefore, the purpose of this study was to examine the long-term test-retest reliability of isokinetic and isometric PT of the leg extensors with a focus on the PT variable determination using several different PT score selection approaches. We hypothesize that reliability will be generally good, but that it will vary considerably based on the PT selection variable, and that there will be a difference in PT values among the different PT selection methods.

## 2. Materials and Methods

Thirteen college-age men (*n* = 8) and women (*n* = 5) volunteered to participate in this investigation (mean ± SD: age = 19.54 ± 1.51, height = 175.11 ± 5.06 cm, mass = 78.93 ± 12.71 kg). The participants were all members of a university club water polo team. Study eligibility criteria required participants to be: (1) between 18–30 years of age, (2) currently a member of the men’s or women’s club water polo team, and (3) free from any lower body injury or surgery within 6 months of the study and any neuromuscular disease. The participants were also not allowed to perform any lower-body resistance training during the study. The study took place during the competitive season where they were involved in 2–3 practices and an average of one water polo game per week [16]. The sample size was determined based on the sample sizes of prior Biodex PT reliability studies. For instance, Lund et al. [8] investigated the Biodex-derived PT variable on both reliability and learning effects over a medium-term period and showed that a sample size of *n* = 13 was sufficient for their study’s purposes, which was similar to this study in terms of design and sample size.

The study was approved by the University Institutional Review Board (#9755; approved 29 January 2019) and all participants read and signed an informed consent document. This study uses a subset of the data collected in a previously published study [16], but the majority of the data herein was not reported previously and this study examines a different research question.

The study involved subjects reporting to the lab for testing on two occasions (trials 1 [T1] and 2 [T2]), separated by a 4-week period. This 4-week period was intended to reflect a period of time that may be implemented in a longitudinal trial in order to examine the reliability of PT measures as would be reflected during an intervention period. There was no intervention given during this period and participants were instructed to maintain their usual routine with as little change as possible. Height and weight were taken on the first visit using a wall stadiometer and calibrated scale (Tanita WB-100A, Tanita Corp., Arlington Heights, IL, USA), respectively. For each testing occasion, participants performed a standardized 5 min warm-up on a cycle ergometer at 50 W, rested for 2 min, and then performed four countermovement jumps with a 1 min rest between jumps. They then performed two warm-up sets of five reps at 50% and 75% of perceived maximum effort with a flywheel squat exercise (kBox4 Pro, Exxentric, Bromma, Sweden), followed by three sets of four maximal repetitions each separated by a 2 min rest. This routine has been previously reported prior to Biodex strength testing [16].

After a 5 min rest period, participants were seated on a Biodex dynamometer (Biodex System 3, Biodex Medical Systems, Shirley, NY, USA) where they were secured to the machine with straps placed over their thigh, chest, and waist in accordance with our previously reported procedures [16]. The center axis of the dynamometer head was set to the knee axis using the investigator’s finger, placed in the direct line between the head and the knee axis. 

The testing was performed on the dominant leg [24]. To acquaint participants with the Biodex leg extension movement, participants performed a localized (thigh region) warm-up of 10 submaximal leg extension/flexion repetitions at ~75% of their perceived maximum at a velocity of 150 deg/s [17,25]. For the testing protocol, participants performed maximal voluntary isometric contractions (MVICs) of the leg extensors for three sets of one repetition, each separated by a 1 min rest. The isometric tests were conducted at a leg angle of 60° below the horizontal plane [19,26]. Following a 1 min break, the participants then performed three sets of three consecutive reps [20] of maximal voluntary isokinetic contractions (MVCs) using concentric/concentric mode through an 80° range of motion [4,16] with each set being separated by a 1 min rest. This was carried out with two velocities, 60 deg/s and 240 deg/s [6,16,18,19], to represent both slow (more force-based) and fast (more velocity-/power-based) contraction velocities [6], respectively. The order of testing always involved the isometric tests first, and then the two isokinetic velocities were randomly determined.

The data sampling and processing procedures have been described in our previous report [16]. Briefly, the raw torque signal (V) was sampled at 2000 Hz with a Biopac data acquisition system (MP150, Biopac Systems Inc., Santa Barbara, CA, USA) and subsequently processed offline with custom-written software (LabVIEW 2018, National Instruments, Austin, TX, USA). The signal was scaled (Nm) and filtered using a zero-phase shift, fourth-order Butterworth filter with a 150 Hz low-pass cut-off frequency [16,27]. The isokinetic torque signal was also corrected for the effect of limb weight/gravity in accordance to the procedures of Aagaard et al. [28]. From the torque-time MVC curves, the isokinetic PT value was calculated as the mean value of the highest 25 ms epoch [16] for the isokinetic 60 deg/s (Isok60) and 240 deg/s (Isok240) contractions. Isometric PT was calculated as the highest 500 ms epoch across the isometric torque-time curve [29].

From the isokinetic data, the participant’s “best score” (i.e., best representative PT value) was calculated using seven different “score selection” methods in order to examine how the diverse score selection approaches—as derived from the nine total repetitions (three sets of three repetitions)—may yield differing PT values. The 7 score selection approaches include the following for both the Isok60 and Isok240 conditions: (1) the single highest rep from set 1 only (Set1Highest), (2) dropping the lowest score in set 1 and taking the mean of the highest two reps from set 1 only (Set1Highest2), (3) the mean of all three reps in set 1 only (Set1Mean3), (4) the mean of the highest of any two reps from the first two sets only (Highest2of2sets), (5) the highest score from all nine reps (i.e., the highest single score of all sets and reps; Highest9), (6) the mean of the highest rep score from each of the three sets (High3setMean), and (7) the mean of all nine sets and reps (Mean9). Finally, for the isometric PT (IsomPT) score selection, there were three different approaches used for calculating the “best” MVIC score: (1) highest score of the three sets (i.e., three sets of one rep; IsomHighest), (2) dropping the lowest score of the three sets and taking the mean of the highest two sets (IsomHighest2), and (3) the mean of all three sets (IsomMean).

### Statistical Analyses

A repeated measures ANOVA was performed to examine the systematic error between T1 and T2 for all variables. The relative consistency (i.e., test-retest reliability) was evaluated from the ICC statistic and the absolute consistency was evaluated from the SEM statistic [30]. Additionally, the minimal difference (MD) needed to be considered real statistic was reported [30,31]. The ICCs were calculated using the “2,1” model from Shrout and Fleiss [32] given this model can be generalized to other laboratories and testers [30,31,33].

Three separate one-way repeated measures ANOVAs were performed across the 7 score selection variables for the Isok60, Isok240, and IsomPT contraction parameters. Post-hoc comparisons used Bonferroni-corrected paired samples *t*-tests. An alpha level of *p* ≤ 0.05 was used to determine whether the outcomes were statistically significant.

## 3. Results

The normality of the data was assessed visually, via the use of plots and deemed to be satisfactory for the variables in the study.

The time between T1 and T2 was 28.8 (SD = 1.8, range = 26–34) days. Table 1 presents the reliability statistics for all PT variables. For the Isok60 velocity, there was no systematic error (*p* = 0.10–0.98) for any of the variables and the ICCs ranged from 0.48–0.81. For the Isok240 velocity, a systematic error was present for Set1Highest2 (*p* = 0.05), Set1Mean3 (*p* = 0.05), and Mean9 (*p* = 0.03) and the ICCs ranged from 0.77–0.87. For the IsomPT variables, a systematic error was present for IsomHighest (*p* = 0.02) and IsomHighest2 (*p* = 0.03) and the ICCs ranged from 0.71 to 0.73.

**Figure 1:**
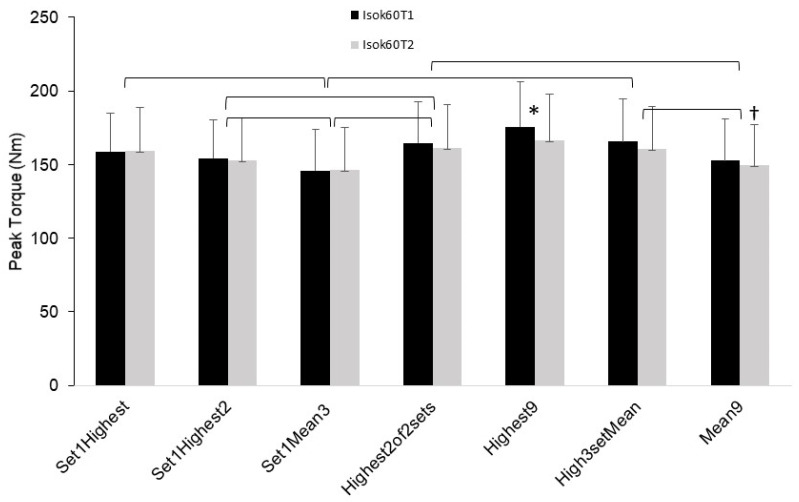
Mean (error bars are SD) peak torque (PT) values for the isokinetic 60 deg/s (Isok60) testing condition for trials 1 (T1) and 2 (T2) for each of the PT score selection parameters. * indicates higher than all other conditions. † indicates lower than “Set 1Highest” condition only for Isok60T2. Isok60 trials 1 and 2 showed the same results (bracket symbols combine the two trials with respect to denoting significance) in all cases except one (noted by †).

For the repeated measures ANOVAs, results for the Isok60 velocity showed there was a significant main effect for both T1 (*p* < 0.001) and T2 (*p* < 0.001) trials. The post hoc comparisons revealed that several differences were present between the conditions and these differences were the same for T1 and T2, with the only exception being between Mean9 and Set1Highest for T2 (*p* = 0.04) which was not significant for T1 (*p* = 1.0). For both T1 and T2, Highest9 was higher than all other conditions (*p* ≤ 0.03 and *p* < 0.02 for T1 and T2, respectively). Set1Highest was higher than Set1Mean3 (*p* < 0.01 and 0.03). Set1Highest2 was higher than Set1Mean3 (*p* < 0.01) and lower than Highest2of2sets (*p* = 0.01 and 0.03). Set1Mean3 was lower than Highest2of2sets (*p* < 0.01) and lower than High3setMean (*p* < 0.01 and 0.02). High3setMean was higher than Mean9 (*p* < 0.01). These results are displayed in Figure 1.

**Figure 2:**
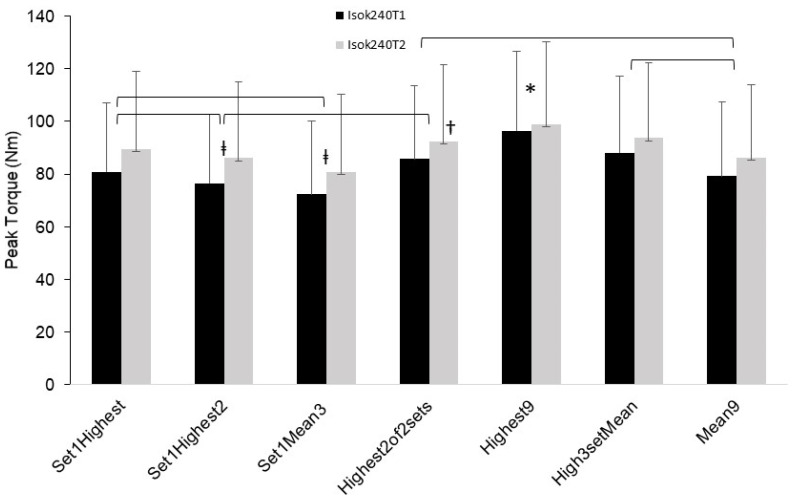
Mean (error bars are SD) peak torque (PT) values for the isokinetic 240 deg/s (Isok240) testing condition for trials 1 (T1) and 2 (T2) for each of the PT score selection parameters. ‡ significantly different from all other conditions except for the “mean9” condition”. † significantly different from “High3setMean” condition only for Isok240T2. * indicates significantly higher than all other conditions except for “Set1Highest” condition for both trials 1 and 2, and the “Highest2of2sets” condition for Isok240T1 only. Isok240 trials 1 and 2 showed the same results (bracket symbols combine the two trials with respect to denoting significance) in all cases except as noted by † and * cases.

Results for the Isok240 velocity showed there was a significant main effect for both T1 (*p* < 0.001) and T2 (*p* < 0.001). The post hoc comparisons revealed that a number of differences were present between the conditions and these differences were mostly the same for T1 and T2, with the exceptions being that Highest2of2sets was lower vs. Highest9 (*p* = 0.05) for T2 but not for T1 (*p* = 0.18). For both T1 and T2, Highest9 was higher than all other conditions except for Set1Highest (*p* = 0.10 and 0.17, T1 and T2, respectively) and Highest2of2sets for T1 only (*p* = 0.18). Set1Highest was higher than Set1Highest2 (*p* = 0.01 and 0.04) and Set1Mean3 (*p* < 0.01). Set1Highest2 was higher than Set1Mean3 (*p* < 0.01). However, Set1Highest2 was lower than Highest2of2sets (*p* = 0.01 and 0.02), Highest9 (*p* = 0.02 and 0.01) and High3setMean (*p* = 0.03 and 0.02). High3setMean was higher than Mean9 (*p* < 0.001). These results are displayed in Figure 2.

**Figure 3:**
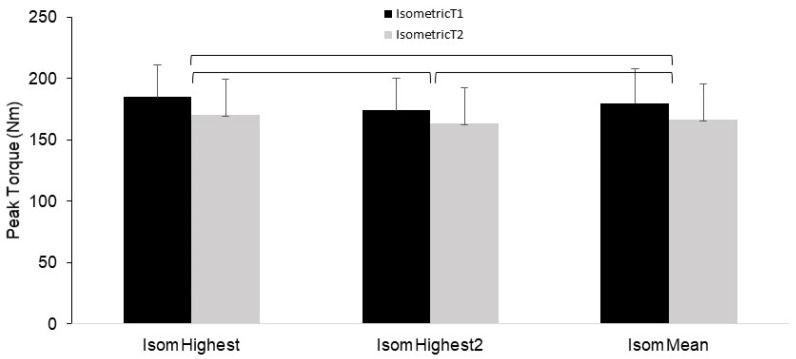
Mean (error bars are SD) peak torque (PT) values for the isometric testing condition for trials 1 (T1) and 2 (T2) for each of the PT score selection parameters. Significance bars (brackets) denote that all conditions are different from each other, with isometric trials 1 and 2 showing the same results in all instances.

Results for the IsomPT variables showed there was a significant main effect for both T1 (*p* < 0.001) and T2 (*p* = 0.001). The post hoc comparisons revealed the differences were all the same for the T1 and T2 trials. For both T1 and T2, all sets were different from each other, such that IsomHighest was higher than IsomHighest2 (*p* < 0.01) and IsomMean (*p* = 0.01) and IsomHighest2 was higher than IsomMean (*p* < 0.01). These results are displayed in Figure 3. Supplementary Table S1 presents the mean and SD values for all the isokinetic and isometric PT variables.

## 4. Discussion

The results of this study demonstrated that the reliability varied widely among the PT variables according to the contraction type and PT score selection method. The different PT selection methods also yielded significantly different PT values for all contraction types.

At the general level, the reliability values were lower than what has been reported previously for the Biodex-derived PT variable [5,8,23]. In the present study, the ICCs ranged from 0.48 to 0.81 for the Isok60 contraction, from 0.77 to 0.87 for Isok240, and from 0.71 to 0.77 for IsomPT; and these ICCs varied markedly according to the PT selection method (Table 1). For the Isok60 contraction, the poorest reliability was found in the mean of the three reps from the set 1 variable (Set1Mean3; ICC = 0.48, SEM = 20.9), along with the mean of the highest two reps from set 1 (Set1Highest2; 0.61 and 17.6) and the highest single rep from set 1 (Set1Highest; 0.67 and 16.4) variables also showing moderately poor reliability. On the contrary, for Isok60, the most reliable variables were the highest single rep of all three sets (Highest9; 0.79 and 13.3), the mean of the highest rep of each of the three sets (High3Mean; 0.81 and 12.6), the mean of all nine reps across all sets (Mean9; 0.78 and 13.3), and the highest two reps of the first two sets (Highest2of2sets; 0.77 and 13.9) variables. Overall the reliability was generally higher for the Isok240 velocity condition. For Isok240, the least reliable of this condition’s variables were the highest two reps of set 1 (Set1Highest2; 0.77 and 11.0), and the most reliable were the highest 2 reps of the first two sets (Highest2of2sets; 0.87 and 8.2), highest single rep of all three sets (Highest9; 0.85 and 8.4), highest rep of each of the three sets (High3setMean; 0.87 and 7.8), and mean of all nine reps across all sets (Mean9; 0.85 and 7.3) variables. The IsomPT contraction condition showed only moderately good reliability such that the PT for the highest single set was the least reliable (0.71), and the mean of the three sets was the most reliable (0.77) of the three IsomPT parameters.

Systematic bias was present in 6 of the 17 conditions. For the Isok240 contraction, systematic bias was found for the mean of the highest two reps from set 1 (Set1Highest2), the mean of the three reps from set 1 (Set1Mean3), and the mean of all nine reps across all sets (Mean9) variables. Systematic bias was also shown for the IsomPT HighestSet and Highest2Sets conditions. None of the Isok60 score selection variables showed systematic bias. More specifically, for the Isok240 contraction, the PT was greater for the second trial compared to the first (showing an increase over the 4-week period), whereas the systematic bias for the IsomPT parameters showed that the second trial was lower than the first trial (showing a decrease over the 4-week period). These findings seem to be at odds, given some score parameters for the Isok240 contraction systematically improved in strength while others for the IsomPT decreased in strength. The reason for this discrepancy is unknown but it could be due to a differential interaction of a learning retention factor between the high velocity (Isok240) and high force (IsomPT) contraction types, such that a long-term learning effect could be present for the faster velocity contractions vs. the zero velocity, high force isometric contractions which may not invoke a long-lasting learning effect due to the static nature of the contraction. More research is needed to elucidate any potential mechanisms responsible for this finding.

It is also possible that the systematic bias outcome could be due to some other factor extrinsic to the study (i.e., lifestyle-related). To this point, it should be noted that given the longer inter-trial time frame inherent to this study, the risk for extrinsic study factors to influence the PT outcome measures would be greater than for a short duration inter-trial time frame as is more commonly found in the literature. Thus, the possibility cannot be excluded that the generally lower reliability from this study, and possible greater systematic bias could be due to the uniquely longer length of the inter-trial period. There seems to be some support for this in the limited research done with longer inter-trial time frames for isokinetic PT reliability such that longer-term (>3 weeks) reliability may exhibit lower reliability than short-term inter-trial periods [6,13]. For instance, Kannus [6] reported knee extensor reliability using the CV statistic and showed a relatively higher (16–20%) variability (i.e., worse reliability) than Madsen’s [13] CV-based reliability of 8% that had only 7 days between test trials. Therefore, the specificity of these findings pertaining to long-term inter-trial isokinetic strength testing periods is important to consider when undertaking research studies that exhibit a several-week period between testing trials, such that researchers should be aware of a possible lower reliability and greater propensity for systematic bias in certain PT variables and contraction conditions.

A particularly novel aspect of this study was the results showing significant differences in PT performance values based on the PT score selection method used (Figure 1, Figure 2 and Figure 3). For the isokinetic conditions (Isok60 and Isok240) the highest rep of all 9 reps (Highest9) was overall greater than the other methods, and the mean of all three reps in set 1 was overall the lowest. Indeed, for the isokinetic conditions, there was a 12–25% PT difference between the highest and lowest methods, showing that the method used for the PT value selection matters considerably. Moreover, the optimal score selection method would ideally capture the “true” best performance for the PT variable and would be capable of doing so with the least amount of trials/reps in order to maximize the time and effort efficiency during testing. While the greatest PT value was the highest of the three sets and three reps (Highest9), two other methods also demonstrated comparatively significantly high PT performance results. These were the mean of the highest reps selected from each of the three sets (High3setMean) and the mean of the highest two reps selected from the first two sets only (Highest2of2sets). These two methods also showed similar reliability. Importantly, the Highest2of2sets PT selection method offers a potential efficiency advantage in that it requires only two sets to be performed, whereas the Highest9 and High3setMean methods require conducting three sets. It would therefore appear the Highest2of2sets method may be the best compromise of efficiency, reliability, and accuracy for testing of isokinetic PT of the leg extensors. This differs from the current common practice of isokinetic testing which overwhelmingly uses a single set model of multiple reps (usually one set of three to five reps).

In fact, when examining the means of each of the individual reps across the three sets, the data show that the highest value occurred at the third rep of set 3 for Isok60 (and the second highest is rep 3 of set 2) whereas it occurs at the third rep of set 2 for the Isok240 condition. Notably though, for Isok60 the difference between the third rep of set 3 and the third rep of set 2 is only 1.5% and 0.37% for trials 1 and 2, respectively; whereas the difference between the third rep in set 2 and the third rep in set 1 is a more substantial 7.5% and 3.1% for trials 1 and 2, respectively. Thus, it appears that more than one set is needed to maximize both reliability and performance assessment and that two sets of three reps may perhaps be the best testing scheme to do this. Moreover, the benefit of the multiple set approach is substantiated in this study by examining the data which showed the set 1 PT selection methods overall had lower reliability and performance than the methods that involved at least 2 sets.

It should be noted that this trend was not exhibited for the IsomPT condition, as the first set (of one rep) was the highest for both trials. However, for this isometric condition, the mean of the three sets of one rep showed the best reliability. Thus, to maximize reliability specifically for the IsomPT condition, the mean of three individual sets should be used in the context of this study design. But it should be noted that the context for the study should be considered given the results presented here are specific to a lengthy inter-trial test period of several weeks. If testing is conducted in a shorter time frame, the reliability is likely to be better for all variables, in which case the best single performance (highest single PT value) could be a viable PT parameter to use.

For comparative purposes, the body of research is very limited with respect to the PT score selection aspect of this study. Cramer and colleagues [17] examined two methods for determining the single representative PT value during isokinetic testing of the leg extensors. They compared the peak value of a single set of three reps vs. the mean of all three reps and reported that there was a difference between the two methods for the 60 deg/s velocity but not for the 180 deg/s velocity. Their data showed that rep 1 was the highest of the three reps, particularly for women. This somewhat contradicts the present findings which showed that rep 1 tended not to be the highest PT value, however, their study population was performed in sarcopenic and malnourished older adults, and they did not use a multiple-set model as this study did. Therefore, comparisons are not directly applicable. Finally, it should be noted that this study is limited to a relatively athletic college-aged population and to concentric (specifically at 60 and 240 deg/s velocities) and isometric conditions on a Biodex model dynamometer. Future research is needed to investigate the use of different testing schemes, such as with a different inter-trial period (i.e., >1 month), and/or more sets (>3) and reps (>3) to determine how even greater testing volumes may influence both reliability and PT score selection factors for PT performance determination.

The present study has some limitations that are noteworthy. The study sample was a relatively athletic college-age population that included a gender-mixed sample. Thus, the results of the study are generalizable to this specific population (which could be a potential strength in filling certain literature gaps, particularly when studies are conducted on a similar population); however, these findings should be used with caution as they may not apply in the same way to other population demographics. The study sample was relatively small and so larger studies may be needed to confirm the current findings and to more robustly establish the recommendations as provided based on these data.

## 5. Conclusions

In conclusion, the present study revealed that over the course of a 1 month inter-trial test period, isokinetic PT reliability tends to be lower than is commonly reported for this variable using shorter inter-trial periods, and that the reliability varies widely across contraction type (mainly velocity-dependent) and PT score selection method. The data also showed that the type of PT score selection method provides substantially different PT values. Using the combined data, the interpretation of this study suggests the collective best approach for a long-term inter-trial test period for isokinetic PT that combines the best PT performance selection with reliability (and reduced possibility of systematic bias) in an efficient manner is a two sets of three reps test scheme, where the PT score selection method involves taking the mean of the two highest reps from the two sets (i.e., mean of the two highest values from the six reps across the two sets). For isometric PT, researchers may consider using the mean of three sets (one rep per set) for enhanced reliability when the testing has a long time delay.

## Figures and Tables

**Table 1:** Reliability statistics for the peak torque (PT) selection conditions for the isokinetic at 60 and 240 deg/s velocities and isometric testing.

Variables	*p*-Value	ICC 2,1	SEM	MD
Isok60-Set1Highest	0.92	0.67	16.40	45.45
Isok60-Set1Highest2	0.83	0.61	17.55	48.65
Isok60-Set1Mean3	0.98	0.48	20.92	57.99
Isok60-Highest2of2sets	0.56	0.77	13.88	38.46
Isok60-Highest9	0.10	0.79	13.31	36.89
Isok60-High3Mean	0.34	0.81	12.60	34.91
Isok60-Mean9	0.59	0.78	13.29	36.84
Isok240-Set1Highest	0.07	0.80	11.15	30.91
Isok240-Set1Highest2	0.05	0.77	11.03	30.59
Isok240-Set1Mean3	0.05	0.80	9.86	27.34
Isok240-Highest2of2sets	0.06	0.87	8.17	22.65
Isok240-Highest9	0.44	0.85	8.43	23.35
Isok240-High3setMean	0.09	0.87	7.83	21.70
Isok240-Mean9	0.03	0.85	7.32	20.30
IsomHighest	0.02	0.71	14.06	38.97
IsomMean	0.07	0.77	13.45	37.28
IsomHighest2	0.03	0.73	13.88	38.48

Isok60 = isokinetic knee extension at 60 deg/s; Isok240 = isokinetic knee extension at 240 deg/s; IsomPT = isometric peak torque; see text for a full description of each of the peak torque variables.

## Data Availability

The raw data supporting the conclusion of this study will be made available by the authors upon request.

## Supplementary Materials

The following supporting information can be downloaded at: https://www.mdpi.com/article/10.3390/jfmk8020062/s1.
