# Supplementary material for: Isokinetic Dynamometer Leg Extensor Peak Torque Measurement: A Time-Delayed Reliability and Score Selection Analysis Study
Source: J Funct Morphol Kinesiol. 2023 May 12;8(2):62. doi: 10.3390/jfmk8020062 (PMC10204485; doi:10.3390/jfmk8020062)
Supplement: Supplementary file 1 [file jfmk-08-00062-s001.zip › jfmk-2291745-supplementary.pdf]

Supplementary Table S1: Means (SD) peak torque (PT) values for the isokinetic and isometric contraction conditions for all PT selection method variables for trials 1 and 2.

Action	Variable	Trial 1	Trial 2
Isok60	Set1Highest	158.8 (26.3)	159.4 (29.4)
	Set1Highest2	154.3 (26.1)	152.8 (29.1)
	Set1Mean3	146.0 (27.8)	146.2 (29.3)
	Highest2of2sets	164.6 (27.8)	161.4 (29.4)
	Highest9	175.8 (30.2)	166.4 (31.2)
	High3setMean	165.6 (29.3)	160.7 (28.6)
	Mean9	152.8 (28.2)	149.9 (27.5)
Isok240	Set1Highest	80.8 (28.0)	89.6 (26.6)
	Set1Highest2	76.5 (26.5)	86.1 (24.3)
	Se1Mean3	72.4 (25.9)	80.8 (23.4)
	Highest2of2sets	85.7 (26.6)	92.4 (23.4)
	Highest9	96.4 (20.6)	99.0 (22.5)
	High3setMean	88.0 (23.8)	93.7 (22.8)
	Mean9	79.3 (21.9)	86.3 (21.9)
IsomPT	IsomHighest	184.7 (32.6)	169.9 (27.1)
	IsomMean	173.7 (32.5)	163.4 (28.3)
	IsomHighest2	179.9 (32.7)	166.1 (28.1)

Isok = isokinetic contraction at 60 or 240 deg/s; IsomPT = isometric peak torque. See main text for a description of all PT selection variables. See main Figures for statistical outcomes/designations.
